# CD40 signaling predicts response to preoperative trastuzumab and concomitant paclitaxel followed by 5-fluorouracil, epirubicin, and cyclophosphamide in HER-2-overexpressing breast cancer

**DOI:** 10.1186/bcr1836

**Published:** 2007-12-17

**Authors:** Francisco J Esteva, Jing Wang, Feng Lin, Jaime A Mejia, Kai Yan, Kadri Altundag, Vicente Valero, Aman U Buzdar, Gabriel N Hortobagyi, W Fraser Symmans, Lajos Pusztai

**Affiliations:** 1 Department of Breast Medical Oncology, Unit 1354, The University of Texas M. D. Anderson Cancer Center, 1515 Holcombe Boulevard, Houston, TX 77030, USA; 2 Department of Bioinformatics and Computational Biology, The University of Texas M. D. Anderson Cancer Center, 1515 Holcombe Boulevard, Houston, TX 77030, USA; 3 Department of Medical Oncology, Hacettepe University Institute of Oncology, Sihhiye St, Ankara 06100, Turkey; 4 Department of Pathology, The University of Texas M. D. Anderson Cancer Center, 1515 Holcombe Boulevard, Houston, TX 77030, USA

## Abstract

**Introduction:**

We performed gene expression analysis to identify molecular predictors of resistance to preoperative concomitant trastuzumab and paclitaxel followed by 5-fluorouracil, epirubicin, and cyclophosphamide (T/FEC).

**Methods:**

Pretreatment fine-needle aspiration specimens from 45 patients with HER-2-overexpressing stage II to IIIA breast cancer were subjected to transcriptional profiling and examined for differential expression of various genes and gene sets. The primary endpoint for tumor response was pathologic complete response (pCR). Correlations between pCR and gene expression were sought.

**Results:**

The overall pCR rate was 64%. Age, nuclear grade, tumor size, nodal status, quantitative expression of estrogen and HER-2 receptor mRNA, and HER-2 gene copy number showed no correlation with pCR. Results of gene set enrichment analysis suggested that the lower expression of genes involved with CD40 signaling is associated with a greater risk of residual cancer after the preoperative chemotherapy that includes trastuzumab.

**Conclusion:**

CD40 signaling may play a role in determining response to trastuzumab-plus-T/FEC therapy in patients with HER-2-overexpressing breast cancer.

## Introduction

The amplification of the *HER-2 *gene is associated with shorter disease-free survival (DFS) and overall survival (OS) in patients with early-stage and metastatic breast cancer [1-3]. The anti-*HER-2 *monoclonal antibody trastuzumab (Herceptin; Genentech, Inc., South San Francisco, CA, USA) is an effective treatment for breast tumors with *HER-2 *gene amplification, both alone and in combination with other therapies. Randomized clinical trials have shown that the addition of trastuzumab to cytotoxic chemotherapy improves DFS and OS in patients with metastatic disease [4,5] and as adjuvant therapy [6,7]. However, not all patients with HER-2-overexpressing tumors benefit from trastuzumab therapy [8]. Approximately 30% of *HER-2*-overexpressing metastatic breast tumors show an objective response to single-agent first-line trastuzumab treatment, and most will eventually progress [9-11]. Similarly, as many as 15% of early-stage HER-2-overexpressing breast tumors will relapse despite adjuvant chemotherapy that includes trastuzumab [6,7].

We recently reported results from a clinical trial in which 42 patients with operable HER-2-overexpressing breast cancer were randomly assigned to receive either 6 months of preoperative T/FEC chemotherapy (four cycles of paclitaxel followed by four cycles of 5-fluorouracil, epirubicin, and cyclophosphamide) alone or concomitant with weekly trastuzumab followed by definitive surgery. An interim analysis showed that the patients in the trastuzumab-plus-T/FEC group had a pathologic complete response (pCR) rate of 65% compared with 26% in the T/FEC-alone group (*P *<0.016) [12]. Because of the large and significant difference in pCR rates, the randomized study was closed after these results became available. A single-arm extension study accrued 22 more patients to the trastuzumab-plus-T/FEC arm to further characterize the efficacy and toxicity of the regimen. In the second cohort, the pCR rate was 55% (95% confidence interval [CI], 32% to 76%).

Because the achievement of pCR correlates closely with improved DFS and OS [13,14], we were interested in examining the clinical and molecular tumor characteristics in the subset of women with residual disease (RD) in our original and extension trials. Our goal was to identify molecular predictors of resistance to preoperative concomitant trastuzumab and T/FEC by analyzing gene expression in specimens from pretreatment fine-needle biopsies. The identification of markers of resistance to trastuzumab-based chemotherapy could help in the design of future clinical trials of novel agents with the potential to overcome drug resistance.

## Materials and methods

### Patients

The therapeutic study and optional biopsy protocol were approved by the institutional review board, and each patient who participated gave written informed consent. Patient accrual and inclusion and exclusion criteria have been described previously [12,15]. Briefly, all patients in both the original and extended trials were required to have histologically confirmed stage II to IIIA, *HER-2*-positive invasive breast cancer. *HER-2 *positivity was defined as the overexpression of HER-2 receptors (an immunohistochemistry [IHC] score of greater than or equal to 3) or *HER-2 *gene amplification (a *HER-2 *gene/centromeric sequence of chromosome 17 [CEP17] ratio of greater than 2, as measured by fluorescence *in situ *hybridization [FISH]). Estrogen receptor (ER) and progesterone receptor (PR) expression levels were measured using IHC.

The initial preoperative regimen consisted of four cycles of paclitaxel at 225 mg/m^2 ^given as a 24-hour infusion at 3-week intervals followed by four cycles of FEC. Patients treated on the open-label phase of the protocol received 80 mg/m^2 ^paclitaxel per week for a total of 12 weeks. The FEC regimen consisted of 500 mg/m^2 ^5-fluorouracil on days 1 and 4, 500 mg/m^2 ^cyclophosphamide on day 1, and 75 mg/m^2 ^epirubicin on day 1. Patients also received a loading dose of intravenous trastuzumab (4 mg/kg over the course of 90 minutes) on day 1 of the first cycle of paclitaxel. Then, patients received 2 mg/kg trastuzumab, administered intravenously over the course of 30 minutes weekly, during each week in which chemotherapy agents were given. All patients underwent mammograms and ultrasound imaging at baseline and during therapy. If, during the course of treatment, imaging detected that a tumor had shrunk to less than 2 cm, metallic markers were placed in the tumor bed under ultrasonographic guidance.

At the completion of preoperative chemotherapy, all patients had a modified radical mastectomy or lumpectomy and a sentinel lymph node biopsy or axillary lymph node dissection, depending on patient preference and the opinion of the surgeon. Grossly visible residual cancer was measured, and representative cross-sections of the tumor were submitted for histopathologic examination. When grossly visible residual cancer was not found, the slices of the surgical specimen were radiographed, and all areas of radiologically or architecturally abnormal tissue were submitted for histopathologic study. A pCR was defined as no residual invasive cancer in the breast and lymph nodes. Residual ductal carcinoma *in situ *was allowed because it has no impact on long-term survival [16].

### Tissue processing and microarray analysis

Patients who participated in the original trial and its extension were given the option of undergoing a one-time, pretreatment fine-needle aspiration (FNA) of their primary tumor. Forty-five patients consented to the procedure. Each FNA was performed using a 23- or 25-gauge needle. Cells from two or three passes were collected into vials containing 1 mL of RNA *later *solution (Ambion, Inc., Austin, TX, USA) and stored at -80°C. RNA was extracted from the FNA samples using the RNAeasy kit (Qiagen Inc., Valencia, CA, USA). The amount and quality of RNA were assessed with a DU-640 spectrophotometer (Beckman Coulter, Fullerton, CA, USA) and considered adequate for further analysis if the optical density 260/280 ratio was greater than 1.8 and the total RNA yield was greater than 1 μg. All FNA specimens met these quality control criteria and were hybridized to Affymetrix U133A GeneChips (Affymetrix, Santa Clara, CA, USA) as described previously [17]. Expression data were quantified using dCHIP version 1.3 (available from [18]). All hybridization experiments met the quality control standards of the study, with scaling factors within threefold of one another, percentage present call rates greater than or equal to 35%, percentage of probe set outliers less than or equal to 15%, percentage of single-probe outliers less than or equal to 5%, and 3':5' ratio less than 3. All gene expression data were normalized to a standard reference chip using the dCHIP software. The normalized gene expression values were transformed to a log_10 _scale for further analysis.

### Statistical analyses

Data analysis was performed using the statistical software package S-PLUS (version 7; Insightful Corporation, Seattle, WA, USA). We used univariate and multivariate logistic regression analyses to examine associations between RD, pCR, and clinical pathologic variables, including routine ER and PR status, Black's modified nuclear grade, tumor size, and level of *HER-2 *gene amplification. To examine correlations between the pathologic response to treatment and the mRNA expression levels of single-gene molecular markers, including ER, Ki67, and HER-2, we performed rank correlation tests as well as unequal variance *t *tests. The probe sets that targeted the most 3' end of each transcript were selected to represent these genes of interest (ER: 205225_at, Ki67: 212021_s_at, and *HER-2*: 216836_s_at). We have previously shown that these probes correlate closely with clinical receptor status but represent more quantitative measurements [19].

We used unequal variance *t *tests to identify genes that were differentially expressed in specimens from patients with RD and those with pCR. Beta uniform mixture analysis of the *P *values was used to estimate false discovery rates (FDRs) [20]. We also examined whether a 30-gene pharmacogenomic predictor of pCR previously developed by our group in patients treated with T/FAC (in which doxorubicin [A] replaces the epirubicin [E] in T/FEC) [17] would predict pCR in patients treated with T/FEC plus trastuzumab. The code for this predictor is available at [21].

We used gene set enrichment analysis (GSEA) to assess the association between pCR, RD, and 1,275 distinct *a priori*-defined gene sets. All of these gene sets are available to the public and have been described in detail in a previous publication [22]. The goal of the GSEA was to determine whether members of a particular gene set (that is, a list of 15 to 500 biologically interesting genes) tended to occur at the top or bottom of the complete rank-ordered gene list. In this case, we ranked the 22,283 probe sets measured by the U133A gene chip based on the probe sets' correlation with pCR. We examined three groups of gene sets, including 319 gene sets corresponding to probes associated with 295 different cryptogenic bands on 24 chromosomes, 522 gene sets corresponding to genes involved in various metabolic and signaling pathways, and 427 gene sets of expression neighborhoods centered on cancer-related genes [22]. Complete linkage hierarchical clustering was carried out using 1,792 probe sets described as the 'revised intrinsic gene set' (supplementary Table 1) and using a 1 – Pearson correlation coefficient [23].

## Results

**Figure 1:**
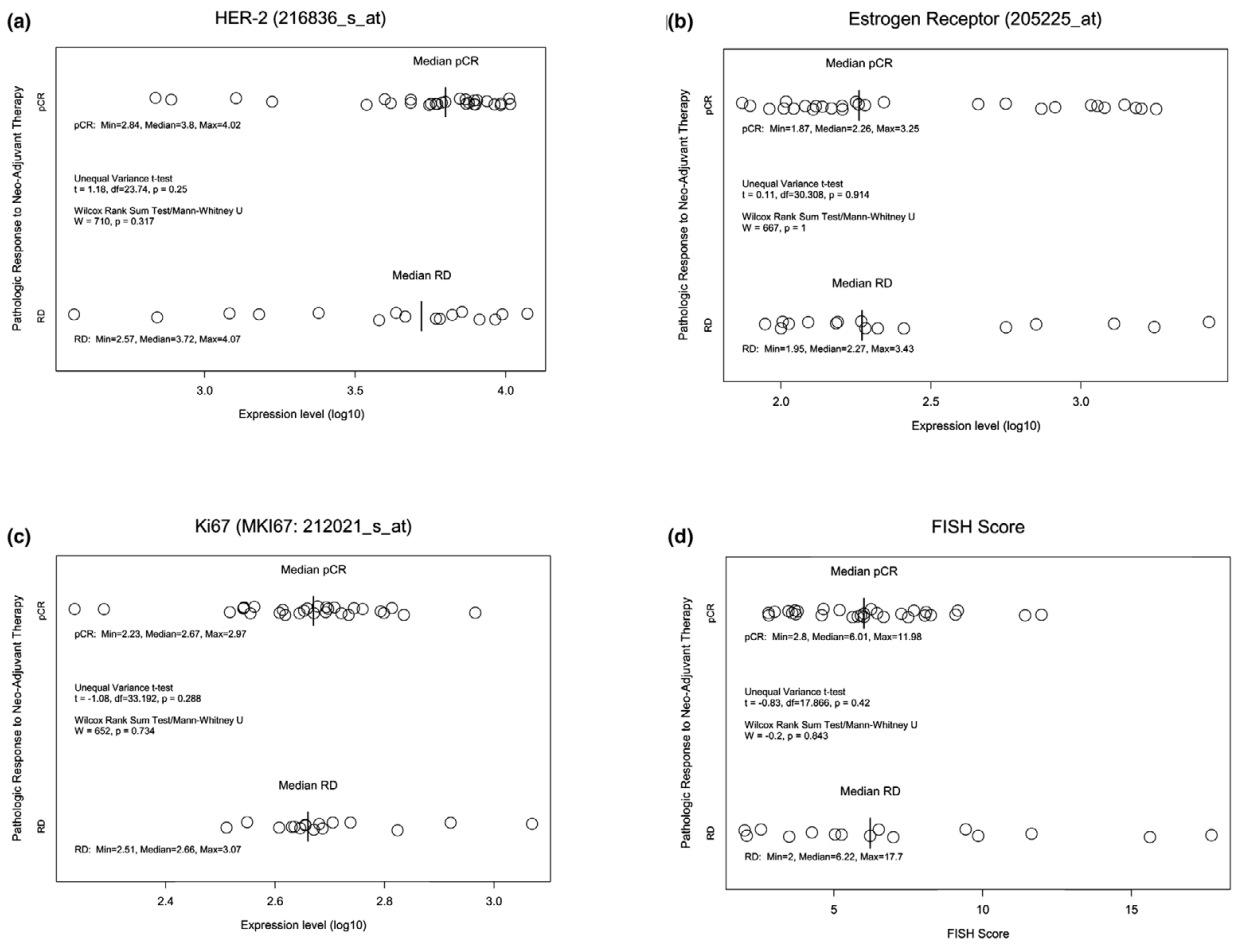
Association between pathologic complete response (pCR) and HER-2, estrogen receptor (ER), and Ki-67 mRNA levels and *HER-2 *gene copy number ratio shown by fluorescence *in situ *hybridization (FISH). Association between pCR and **(a) **the mRNA expression levels of HER-2 (Spearman's rank correlation = 0.087; *P *= 0.69), **(b) **ER (Spearman's rank correlation = -0.052; *P *= 0.81), and **(c) **Ki-67 (Wilcoxon rank sum = 148; *P *= 0.2) or **(d) **the level of *HER-2 *gene amplification shown by FISH (Wilcoxon rank sum = -0.45; *P *= 0.65). Each circle represents results from one case and has been grouped by response category (pCR or RD [residual disease]).

**Figure 2:**
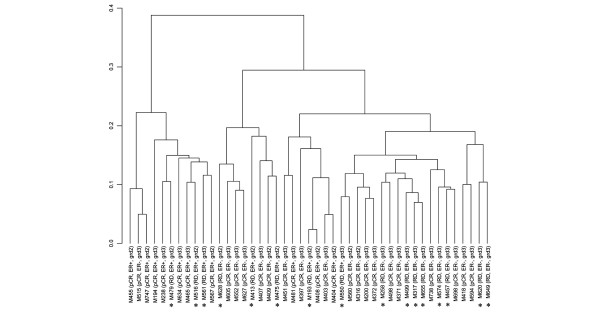
Hierarchical clustering with the revised intrinsic gene set. Supervised cluster analysis using 1,792 intrinsic probe sets showed two distinct clusters among these HER-2-positive cases, but the clusters were not associated with known clinical variables or response to therapy. Cases with residual disease (RD) are marked with an asterisk. ER, estrogen receptor; pCR, pathologic complete response.

Forty-five women who received concomitant trastuzumab and T/FEC volunteered for pretreatment FNA biopsies. Patient and disease characteristics are presented in Table 1. Twenty-nine patients (64%) achieved pCR after 24 weeks of preoperative therapy. Age, nuclear grade, tumor size, nodal status, clinical ER status, and *HER-2 *gene amplification level, as detected by FISH, showed no significant association with pCR in either univariate or multivariate logistic regression analyses. Quantitative expression of the ER and *HER-2 *receptor and Ki67 mRNA levels also showed no significant association with pCR in univariate analyses (Figure 1). Supervised cluster analysis using the revised intrinsic probe sets showed two distinct clusters, but these were not associated with known clinical variables or with response to therapy (Figure 2). An unequal variance *t *test identified 132 genes with *P *values of less than 0.01 which were seemingly differentially expressed between cases of pCR and RD. However, the FDR associated with these *P *values was near 100%. Therefore, we could not identify any genes that would discriminate between pCR and RD with statistical confidence.

**Table 1:** Patient and tumor characteristics

Characteristic	Number of patients (percentage)
Age	
<50 years	19 (42)
≥50 years	26 (58)
*HER-2*^a^	
IHC (score 3+)	26 (58)
FISH-positive	42 (95)
Estrogen receptor-positive	
Yes	17 (38)
No	28 (62)
Progesterone receptor-positive	
Yes	10 (22)
No	35 (78)
Nuclear grade	
Intermediate	14 (31)
High	31 (69)
Tumor size (baseline)	
T1	5 (11)
T2	24 (53)
T3	8 (18)
T4	8 (18)
Nodal status (baseline)	
N0	19 (42)
N1-N3	26 (58)

^a^All tumors were HER-2-positive by either IHC (score, 3+) and/or FISH. Not every patient had both tests. Two tumors were 2+ by IHC and positive by FISH, and one tumor was 3+ by IHC and negative by FISH. FISH, fluorescence *in situ *hybridization; IHC, immunohistochemistry.

We also tested the accuracy of a 30-gene pharmacogenomic predictor of pCR which had been previously developed from a study of patients treated preoperatively with T/FAC. This predictor showed both high sensitivity (92%) and negative predictive value (NPV) (96%) in an independent validation conducted in a cohort of T/FAC-treated patients [17]. The same 30-gene test showed a diminished sensitivity of 52% (95% CI, 0.27 to 0.79) for predicting pCR in patients treated with trastuzumab plus T/FEC. The NPVs and positive predictive values were also low at 36% (95% CI, 0.17 to 0.59) and 65% (95% CI, 0.43 to 0.84), respectively. The overall accuracy was 51% (95% CI, 0.36 to 0.66) in the present cohort of patients compared with 76% (95% CI, 0.62 to 0.87) in the validation cohort, which did not receive trastuzumab.

However, because the original validation cohort included both HER-2-positive and HER-2-negative tumors and the cohort of the present study included only HER-2-positive tumors treated with trastuzumab, it was unclear whether the diminished accuracy was due to the inclusion of trastuzumab or the application of the predictor. We therefore calculated the performance metrics for the subset of patients in the validation cohort who had HER-2-positive tumors and compared them with the results from the present study and found that overall accuracy, sensitivity, and specificity remained high in this group of patients (Table 2).

**Table 2:** Predictive performance (and 95% confidence interval) of the DLDA30 predictor in patients treated with preoperative chemotherapy

	Breast tumors of any phenotype	HER-2-positive breast tumors	
	
Performance metrics	Validation cohort T/FAC^a^ *n *= 51	Present study cohort Trastuzumab + T/FEC *n *= 45	Subset of validation cohort T/FAC^a^ *n *= 30
Area under the curve	0.877 (0.066)	0.565 (0.089)	0.81 (0.092)
Overall accuracy	0.76 (0.62–0.87)	0.51 (0.36–0.66)	0.67 (0.47–0.83)
Sensitivity	0.92 (0.64–1.00)	0.52 (0.33–0.71)	0.90 (0.56–1.00)
Specificity	0.71 (0.54–0.85)	0.50 (0.25–0.75)	0.55 (0.32–0.77)
Positive predictive value	0.52 (0.31–0.73)	0.65 (0.43–0.84)	0.50 (0.26–0.74)
Negative predictive value	0.96 (0.82–1.00)	0.36 (0.17–0.59)	0.92 (0.62–1.00)

^a^Hess KR, Anderson K, Symmans WF, Valero V, Ibrahim N, Mejia JA, Booser D, Theriault RL, Buzdar AU, Dempsey PJ, *et al*. [17]. T/FAC, paclitaxel followed by 5-fluorouracil, doxorubicin, and cyclophosphamide; T/FEC, paclitaxel followed by 5-fluorouracil, epirubicin, and cyclophosphamide.

**Figure 3:**
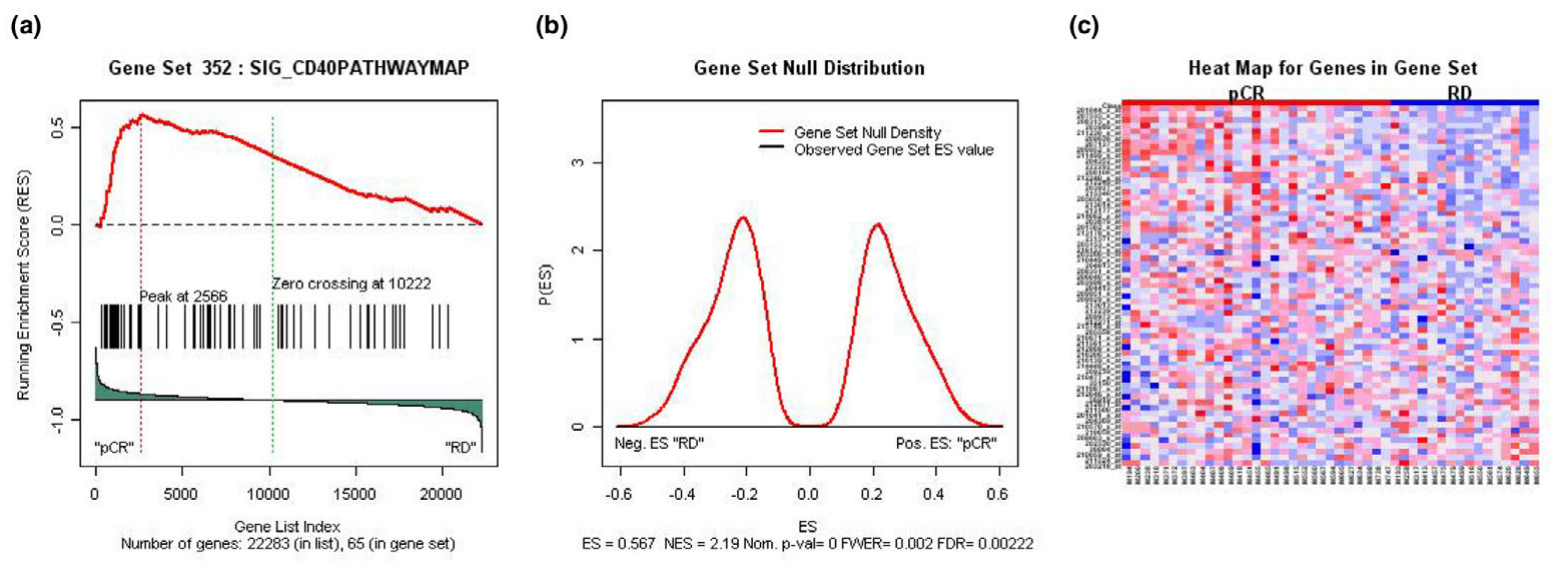
CD40 pathway gene set significantly enriched in patient's pathologic complete response and residual disease phenotypes. **(a) **Plot of the running sum for CD40 pathway gene set in the expression data set. **(b) **Plot of null bimodal ES distributions **(c) **Heat map produced using expression data from CD40 pathway gene set, and sorted by pCR and RD phenotypes. ES: enrichment score; FDR: false discovery rate; FWER: family wise error rate; NES: normalized enrichment score; pCR: pathologic complete response; RD: residual disease.

The evaluation of 1,275 distinct gene sets showed significant enrichment for only one gene signature: the CD40 signaling pathway, which involves 64 genes (supplementary Table 2). Complete results from the GSEA are included in supplementary Table 3. Patients whose tumors expressed the CD40 signature were significantly more likely to achieve pCR. The FDR associated with this observation was 0.0022 (Figure 3).

## Discussion

This analysis of gene expression data, which included 22,277 probe sets corresponding to more than 14,000 genes, revealed no robust gene expression differences between the two subgroups of HER-2-positive patients (those with pCR and those who had RD) treated with preoperative concomitant trastuzumab and T/FEC. Relatively modest differences in gene expression can have important biological implications if the number of genes is large enough; however, the transcriptional differences between the two response categories tested here were subtle and thus not easily identified using *t *statistics. Therefore, we subjected our data to GSEA, a more complex analytical method than *t *statistics, which can detect subtle gene expression differences for *a priori*-defined sets of genes between two groups (for this study, patients with pCR and those with RD). GSEA results indicated that patients whose tumors showed decreased expression of the genes associated with the CD40 signaling pathway were more likely to have residual cancer after trastuzumab-plus-T/FEC chemotherapy. One of the reasons why we could not find more differentially expressed gene sets may be the small sample size and the low number of patients in the group who had RD after preoperative chemotherapy and trastuzumab treatment. It should be noted that, in small pharmacogenomic studies, the association between large-scale gene expression patterns and ER status and grade represents substantial biological noise for outcomes that are not associated with these clinical variables [24].

*HER-2 *gene amplification (*HER-2*/CEP17 ratio of greater than 2.0) and protein overexpression (IHC staining intensity of greater than 3) remain the best predictors of response to trastuzumab therapy [2,4]. According to these criteria, all patients in the present study were *HER-2*-positive. We found no correlation between the *HER-2 *gene copy number or semiquantitative *HER-2 *mRNA levels and the amount of residual invasive cancer after preoperative T/FEC-plus-trastuzumab therapy. This suggests the existence of a threshold effect, in which a *HER-2*/*CEP17 *ratio of greater than 2.0 would predict a response to trastuzumab.

There are many research findings indicating the potential oncogenic role of CD40. In particular, although CD40, a member of the tumor necrosis factor receptor family, is expressed in B lymphocytes, macrophages, fibroblasts, and endothelial and epithelial cells, it is also expressed in a variety of hematologic malignancies and solid tumors. For example, transfection studies have shown that constitutive CD40 signaling plays an important role in the transformation of murine fibroblasts, an oncogenic effect that was inhibited by the suppression of the nuclear factor-kappa B signaling pathway [25]. Similarly, the treatment of human breast cancer cells with a recombinant CD40 ligand (CD40L) inhibited cell growth *in vitro *and *in vivo *[26,27]. Wingett and colleagues [28] observed that, in contrast to normal B cells, in which CD40 signaling provides a potent survival signal, CD40 ligation in breast carcinoma cells results in growth inhibition and enhanced susceptibility to Fas-mediated apoptosis. Tong and colleagues [29], in their study using CD40-positive (T47D and BT-20) and CD40-negative (MCF-7 and ZR-75-1) cell lines, likewise examined the growth outcome of CD40 ligation in human breast cancer cells. They found that treatment with CD40L reduced [^3^H]thymidine uptake in BT-20 and T47D cells but did not affect the growth of CD40-negative MCF-7 or ZR-75-1 cells. These investigators observed similar results *in vivo*. CD40 expression has also been shown to play a role in the activation of dendritic cells, which are promising candidates for breast cancer immunotherapy [30,31]. In keeping with this finding, Pinzon-Charry and colleagues [32] found a significantly higher proportion of apoptotic circulating dendritic cells in patients with early-stage breast cancer (stage I to II; *n *= 13) than in healthy volunteers (*n *= 15). These findings collectively show the oncogenic potential of CD40.

Several preclinical studies from different laboratories have suggested that the CD40 signaling pathway plays a role in the modulation of chemosensitivity in breast cancer cells. In one such study, Stumm and colleagues [33] reported that stimulation of the CD40 receptor inhibited paclitaxel-induced apoptosis in breast cancer cell lines. Voorzanger-Rousselot and colleagues [34] showed that, in breast cancer cell lines, doxorubicin, cisplatin, etoposide, vinblastine, and paclitaxel increased apoptosis in a dose-dependent manner through a caspase-independent mechanism. Co-culture with irradiated L cells expressing CD40L significantly reduced the percentage of apoptotic cells in breast cancer cell lines treated with these drugs. These experimental data are consistent with our observation that the activation of the CD40 pathway in primary breast tumors is associated with an improved pathologic response to preoperative trastuzumab-plus-T/FEC chemotherapy.

To address the potential problem of the characteristics of our validation cohort not sufficiently matching those of the present study cohort, we examined the predictive performance of a previously developed 30-gene pharmacogenomic predictor of pCR which had also been validated in patients who received preoperative T/FAC alone, regardless of HER-2 status [17]. The genomic predictor was less accurate in the trastuzumab-treated patients than in the patients of the validation cohort who did not receive trastuzumab. However, the 30-gene predictor also performed well in the HER-2-positive subset of patients included in the original validation set. This suggests that the loss of accuracy was due to the inclusion of a new drug, trastuzumab, rather than the application of the predictor to a particular subset of cases. The lack of correlation between factors known to be predictive of pCR in preoperative chemotherapy trials, such as ER and Ki67, can be explained by the powerful effect of trastuzumab in patients with HER-2-overexpressing tumors.

Our previous work identified alterations in protein levels and protein phosphorylation as important contributors to trastuzumab resistance *in vitro *[35]. Potential mechanisms of trastuzumab resistance include loss of PTEN (phosphatase and tensin homolog deleted on chromosome 10) [36], cross-talk between the HER-2 and insulin-like growth factor-I receptor proteins [37], and downregulation of the p27^kip1 ^protein [38]. Such protein level alterations cannot be directly measured by transcriptional analysis; hence, it is not surprising that these genes did not emerge as predictors from our microarray data set. Protein level analysis of these clinical specimens has yet to be performed. Indeed, it is possible that markers of trastuzumab resistance will be easier to identify at the protein level than at the mRNA expression level.

It is important to point out that, in this study, only 36% of patients (*n *= 16) had residual invasive breast cancer after 6 months of preoperative T/FEC-plus-trastuzumab therapy and that all of these patients had some clinical response to therapy. Therefore, any analysis we can perform can only compare extreme sensitivity (that is, pCR) to lesser sensitivity (that is, clinical response but pathologic RD). This may have limited our ability to identify transcriptional differences between these two response groups. Also, sample size was limited by the availability of specimens, a challenge inherent in most optional correlative science studies. As similar studies are published and more transcriptional data become publicly available, the option to perform pooled analyses may allow investigators to study larger data sets.

## Conclusion

In summary, our findings indicate that certain clinical variables, including grade and ER status, and a pharmacogenomic predictor of pCR which has been proven accurate in the absence of trastuzumab are no longer accurate when trastuzumab is included in the treatment. Our GSEA results suggest that a CD40 gene signature may be able to identify a subset of patients with HER-2-overexpressing breast cancer who are more likely to achieve pCR in response to trastuzumab-plus-T/FEC therapy.

## Abbreviations

CEP17 = centromeric sequence of chromosome 17; CI = confidence interval; DFS = disease-free survival; ER = estrogen receptor; FDR = false discovery rate; FISH = fluorescence *in situ *hybridization; FNA = fine-needle aspiration; GSEA = gene set enrichment analysis; IHC = immunohistochemistry; NPV = negative predictive value; OS = overall survival; pCR = pathologic complete response; PR = progesterone receptor; RD = residual disease; T/FAC = paclitaxel followed by 5-fluorouracil, doxorubicin, and cyclophosphamide; T/FEC = paclitaxel followed by 5-fluorouracil, epirubicin, and cyclophosphamide.

## Competing interests

M. D. Anderson Cancer Center (Houston, TX, USA) has received clinical research funding to conduct clinical trials from Genentech, Inc. (South San Francisco, CA, USA), which is the manufacturer of trastuzumab (Herceptin). The authors declare that they have no competing interests.

## Authors' contributions

FJE conceived of the study, participated in its design and coordination, and helped to draft the manuscript. JW, FL, and KY performed the statistical analysis. JAM and KA participated in the acquisition, analysis, and interpretation of clinical data. VV, AUB, GNH, WFS, and LP participated in the design and coordination of the study and helped to draft the manuscript. All authors read and approved the final manuscript.
